# Development of a System Architecture for Evaluation and Training of Proprioceptive Deficits of the Upper Limb

**DOI:** 10.1155/2018/4132820

**Published:** 2018-01-10

**Authors:** Roberto Colombo, Alessandra Mazzone, Carmen Delconte, Fabrizio Pisano

**Affiliations:** ^1^Service of Bioengineering, IRCCS ICS Maugeri Spa SB, Pavia, Italy; ^2^Service of Bioengineering, IRCCS ICS Maugeri Spa SB, Veruno, Italy; ^3^Neurologic Rehabilitation Division, IRCCS ICS Maugeri Spa SB, Veruno, Italy

## Abstract

Proprioception plays a fundamental role in maintaining posture and executing movement, and the quantitative evaluation of proprioceptive deficits in poststroke patients is important. But currently it is not widely performed due to the complexity of the evaluation tools required for a reliable assessment. The aims of this pilot study were to (a) develop a system architecture for upper limb evaluation and training of proximal and distal sense of position in the horizontal plane and (b) test the system in healthy and pathological subjects. Two robotic devices for evaluation and training of, respectively, wrist flexion/extension and shoulder-elbow manipulation were employed. The system we developed was applied in a group of 12 healthy subjects and 10 patients after stroke. It was able to quantitatively evaluate upper limb sense of position in the horizontal plane thanks to a set of quantitative parameters assessing position estimation errors, variability, and gain. In addition, it was able to distinguish healthy from pathological conditions. The system could thus be a reliable method to detect changes in the sense of position of patients with sensory deficits after stroke and could enable the implementation of novel training approaches for the recovery of normal proprioception.

## 1. Introduction

Most patients after incomplete spinal cord injuries and stroke experience upper and lower extremity impairments that can result in persistent limitations in their activity and participation domains as defined by the International Classification of Functioning, Disability and Health (ICF). The most common impairments are motor deficits such as paraplegia and hemiparesis, which are experienced by a large proportion of patients [1, 2]. Impairment of body sensation following the acute event is common, with a significant proportion of patients experiencing deficits of their proprioceptive abilities [3–6].

Proprioception can be defined as the ability of an individual to perceive body segment position, movement in space, and force generated by the body [7–9]. It is based on sensory signals that muscles, joints, and skin receptors provide to the central nervous system (CNS) consequent upon stretch and compression of body tissue. Because of the important role played by proprioception in maintaining posture and in movement execution and control, patients who exhibit proprioceptive deficits cannot maintain their limbs in a steady posture or execute controlled movements without the support of vision [10]. People suffering loss of proprioceptive feedback move by relying on vision, but long processing delays inherent to the visual system (100–200 ms) yield movements that are typically slow, poorly coordinated, and require a good deal of attention [11]. As a consequence, visually guided corrections may come too late and result in jerky, unstable movements [12]. Sensory function, as well as motor function, is important for dexterity tasks [13]. Therefore, stroke survivors often give up using their impaired limb because of their sensorimotor deficits even though this reduces their quality of life [14].

Proprioceptive deficits can also interfere with motor learning processes, as well as with the motor outcome of rehabilitative treatment and the recovery after neurological injuries [15, 16].

However, sensory rehabilitation is often neglected as clinicians either do not treat the problem or use assessment and treatment methods lacking a sound theoretical or empirical basis [17, 18]. A recent survey reported that about 90% of professional therapists (occupational therapists and physiotherapists) routinely assess for sensory loss but the majority (>70%) of them do not use standardized measures [19]. In addition, evidence-based treatments to address sensory impairment are not common and therapists frequently rely on colleagues' opinions and previous experience to inform practice.

Proprioception on its own is difficult to measure; it is commonly evaluated by clinicians through tests which have poor interrater reliability and sensitivity and give only a qualitative and subjective measure [20, 21]. Different methods have been proposed for the quantitative evaluation of proprioceptive deficits [10, 22–26]. Most of them rely on joint position-matching task procedures in a plane or, alternatively, the ipsilateral and contralateral matching of a joint angle [27].

The most frequently reported treatments target the upper limb and hand and include compensatory strategies, sensory reeducation (e.g., subjective grading of stimuli including/excluding visual feedback), sensory feedback on body sensation in the context of everyday activities, and discrimination of limb position and movement. For example, the sensory discrimination training proposed in the recent Study of the Effectiveness of Neurorehabilitation on Sensation (SENSe study) includes 3 sensory tasks: texture discrimination, limb position sense, and tactile object recognition [5]. Various other passive stimulation approaches have been tested in an attempt to regain lost sensory function and, as a result, recover motor function. They include electrical stimulation [28], intermittent pneumatic compression [29], thermal stimulation [30], and peripheral magnetic stimulation [31]. However, these techniques have been limited to improving tactile and kinesthetic sensation. A recent review paper [32] found that (a) proprioceptive training can improve proprioception and can lead to recovery of somatosensory and sensorimotor functions; (b) improvements depend on the intervention's duration; and (c) a large population may gain benefit from proprioceptive training regardless of the neurological or musculoskeletal origin of the somatosensory deficits.

Robot-assisted neurorehabilitation of the upper limb, thanks to its capacity to deliver high intensity training protocols, has the potential for a greater impact on impairment and motor function both in subacute and chronic stroke [33, 34], and many different devices have been proposed for use in clinical and/or home settings [35].

However, there are few studies related to technology-aided rehabilitation focusing on the improvement of proprioception itself. One example is the study by Cho et al. who used a virtual reality (VR) rehabilitation system to develop an interactive game for upper limb training; by blocking visual feedback in specific phases of the game related motor task, the VR system was able to improve proprioceptive deficits in a group of stroke individuals [36]. Casadio and colleagues [37] proposed a proprioception-based motor training technique to augment kinesthetic awareness via haptic feedback mediated by a robotic manipulandum. Specifically, they alternated blocks of reaching trials performed with and without visual cues. De Santis et al., on the other hand, proposed a method based on robotic training that is effective in enhancing kinesthetic acuity [38]. Recently, our research group successfully tested the application of a conventional robot-assisted training protocol to improve proprioceptive deficits in a subject after chronic stroke [39]. However, no study has yet verified if proprioceptive training of the proximal joints (shoulder and elbow) is able to elicit changes in distal (wrist) proprioception.

The aim of the present paper, therefore, is to present the design concepts and the preliminary testing of a system architecture specifically developed for the evaluation and training of proprioceptive deficits of the proximal and distal upper limb. This architecture should allow (a) the implementation of specific treatment protocols devoted to the rehabilitation of sensory and motor functions of the whole arm and (b) the evaluation of their effectiveness in the recovery of proprioception in different districts (proximal and distal).

## 2. Methods

### 2.1. System Architecture

The system architecture consists of two robotic devices that we routinely use for the rehabilitation of wrist and shoulder-elbow functions. The first one is used mainly for testing of distal proprioception and the latter for training and evaluation of proximal proprioception.

### 2.2. Device for Evaluation of Distal Proprioception

**Figure 1:**
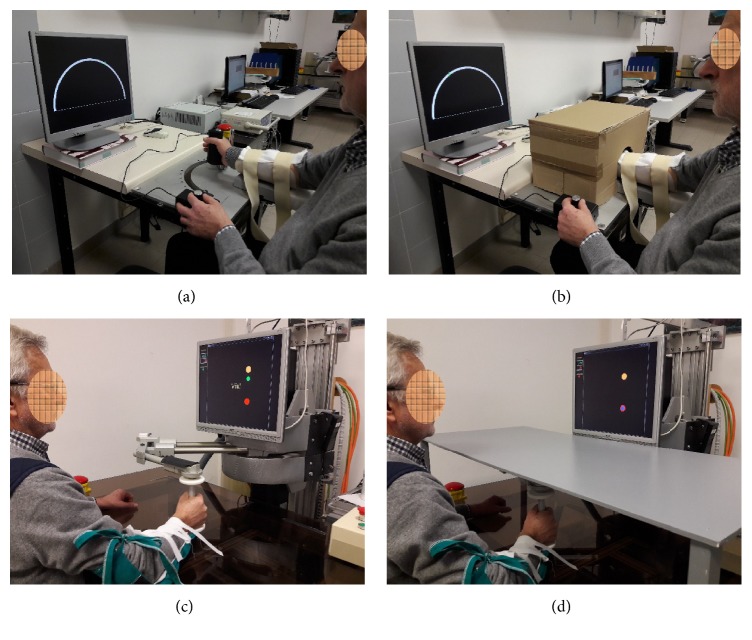
(a) and (b): Device for the evaluation of wrist proprioception. (c) and (d): Device for training and evaluation of proximal proprioception. Of note the two different solutions to prevent use of visual feedback during evaluation and training.

The device is presented in Figure 1(a) and it was previously developed for robot-assisted wrist rehabilitation. It consists of a DC motor fixed to the plane of a specific table. The motor shaft is connected to the handle used to displace the patient's hand so as to obtain wrist flexion or extension movements. Speed and position signals are obtained, respectively, by a tachometer and potentiometer mounted on the motor shaft, which provide feedback signals for the motor control. The system allows a workspace for the patient of −90, 0, and +90 deg. The transducers' analog signals are fed to a personal computer through an analog-to-digital (A/D) interface acquiring data at a 100 Hz sampling rate. The computer both collects data and controls the DC motor. Details of the device have been reported elsewhere [40].

The evaluation procedure is similar to that recently presented by Rinderknecht et al. [41]. The subject is comfortably seated at the robot desk in such a way that the subject's mid-sagittal axis is approximately aligned with the center of the PC monitor displaying a half-circle graded scale, representing the possible range of displacement during flexion/extension. The robot produces a pseudorandom sequence of 33 passive wrist flexions/extensions of the upper limb in the horizontal plane (passive hand) covering predefined positions in the −50, 0, and +50 deg range. The hand and wrist are masked by an opaque box (Figure 1(b)) so as to prevent knowledge of the current position of the passive hand through visual feedback. Then, the subject is requested to move a cursor on the screen, by rotating a potentiometer with the contralateral hand. If the patient is unable to rotate the potentiometer with the impaired arm (for evaluation of the unimpaired arm), only the impaired arm is evaluated. The discrepancy between the actual and estimated positions of the passive hand allows measurement of the sense of position at the wrist joint through a set of quantitative parameters (see Section 2.4). The series of passive rotational wrist displacements increases linearly and movement speed is randomly set at one of three different values (20, 30, or 40 deg/s). The experimental protocol includes also a pretest practice of a short sequence (9 displacement points) of the same task without the masking box so as to allow task learning and exclude the risk of parallax errors due to misalignments between the subject's mid-sagittal axis and the PC screen center.

### 2.3. Device for Training and Evaluation of Proximal Proprioception

The two-DoF elbow-shoulder manipulator “Braccio di Ferro” [42] is used for the treatment and evaluation of proximal proprioception (Figure 1(c)). The end-effector of the robot apparatus consists of a sensorized (position sensor) handle which is grasped by the subject and moved through the workspace of the device (i.e., in the horizontal plane). Subjects are requested to complete a motor task consisting of a sequence of point-to-point reaching movements in the shape of a geometrical figure. The subject is seated facing a video screen that provides visual feedback in the form of three colored circles: (1) a yellow circle indicates the task's starting position; (2) a red circle indicates the target position; (3) a green circle indicates the current position of the handle. Details of the administered tasks and procedures have been extensively reported elsewhere [40, 43].

For training of proprioception, subjects are instructed to move the handle from the starting point to the target of the reaching path with partial assistance of vision. Specifically, the subject's vision of the arm and robot handle is blocked through a specific opaque plane (Figure 1(d)). In addition, visual feedback on the current position of the handle disappears after 5% of the trajectory travelled. When the patient stops movement because he/she estimates to have reached the target, the visual feedback appears for half a second. In this way the robot provides visual assistance and the patient, if able, can restart movement. When the patient stops because they are unable to complete the motor task, the robot drives the arm to the target, providing haptic assistance. At the end of the movement (last 5% of the trajectory), the hand cursor position will briefly reappear and the subject is allowed to make a correction to the handle's final position (vision phase). Again, the robot assists the subject to complete the task if he/she is unable to do so autonomously, by providing a constant assistive force directed toward the target.

### 2.4. Evaluation Parameters

The system and tasks implemented allow evaluation of distal and proximal proprioception through the measurements of a set of quantitative parameters.

Wrist proprioception is evaluated by measuring the discrepancy between the actual position of the displaced (flexed/extended) hand and the estimated position indicated by positioning a cursor on the computer screen through rotation of a potentiometer. The parameters considered are as follows:

- *Mean Error (ME)*: the average value of the differences between actual and estimated position (position error).
- *Absolute Error (AE)*: the average absolute value of the differences between actual and estimated position.
- *Variability (VE)*: the standard deviation of the position error.
- *Error Gain (EG)*: the slope of the regression line between actual (independent variable) and estimated positions (dependent variable).

During training with the shoulder-elbow manipulator, the status of the visual feedback and robot assistance are continuously recorded and can be used to assess the patient's improvement and to indirectly estimate proximal proprioception performance in terms of the following parameters:

- *Average Visual Assistance (VA)*: this is the average percentage of the trajectory (i.e., the number of distance units from the starting point with respect to the total reach distance) at which the subject requested vision assistance. The value is computed as the average value during a training session.
- *Number of Visual Assistance (NVA) Activation*: that is, the number of times that visual assistance was requested during each training session, normalized to the number of reaching movements.
- *Active Movement Index (AMI)*: this index expresses the average percentage of the trajectory completed by the patient using voluntary activity. In other words, it represents the point of trajectory (i.e., the number of distance units from the starting point with respect to the total reach distance) at which the robot assistance is activated [40].

### 2.5. Subjects and Clinical Evaluation Tools

This architecture was tested in a group of healthy individuals and in patients after stroke. In particular, the robot for evaluation of wrist sense of position was applied in a group of 12 right-dominant healthy individuals and in 6 patients after stroke with and without proprioceptive deficits. The limb was placed on a foam support in such a way that it could easily grasp the robot handle; the wrist was in neutral position and the fingers were placed around the handle and fastened by means of a belt. The forearm was fastened to the support in semiprone position by means of two belts so as to allow only flexion or extension of the hand in the horizontal plane.

The device for training and evaluation of proximal proprioception of the upper limb was applied in a separate group of 4 stroke patients in subacute condition with proprioceptive deficits. During training, the patient's paretic limb was supported at the elbow by a low friction pad that slid along the surface of the robot desk. Due to the fact that the implementation of the wrist proprioception evaluation device and the recruitment of the patients trained with the device for proximal joints took place in two different moments of time, we could not assess wrist sense of position in this group of subjects.

The robot treatment was performed in addition to conventional physical therapy. All patients received physical therapy carried out by professionals, without knowledge of the study, according to the Italian Stroke Prevention and Educational Awareness Diffusion (SPREAD) guidelines for 45 min. a day on the same days as robot treatment [7]. Patients were also given two scales at the start and end of treatment by a trained rehabilitation professional: (1) the Fugl-Meyer scale to assess patients' motor (FM-M) and sensory (FM-S) level of impairment; the evaluation was limited to the upper limb section (range = 0–66 and 0–12, resp., for motor and sensory impairment) and (2) the Modified Ashworth scale (MAS) to evaluate muscle tone at the elbow, shoulder, and wrist.

### 2.6. Statistical Analysis

The wrist proprioception parameters were measured in both left and right arms of healthy subjects. Given the small sample size of the healthy group, the difference of performance between arms for each parameter was assessed by the nonparametric Mann–Whitney* U* test for independent samples. In addition, single subject analysis was carried out for the 6 stroke patients evaluated with the wrist device, in order to compare the slope of the regression line obtained in the whole group of healthy subjects with that of each stroke patient. A significance level of 0.05 was adopted for the statistical tests.

The parameters evaluating the performance obtained with the device for training and evaluation of proximal proprioception were computed before and after treatment. Trend in time course of the parameters was assessed by linear regression. Due to the small number of subjects recruited in this study subsection, only a preliminary qualitative analysis was carried out for each subject.

## 3. Results

The demographic and clinical characteristics of the healthy subjects and patients are reported in Table 1.

Table 2 reports the results of the wrist sense of position parameters measured in both left and right arms of the healthy subjects. For each parameter, the mean value, standard deviation, and comparison between left and right arms are reported. Small mean and absolute errors were obtained in both arms. Higher values were found for the right arm than for the left. The comparison showed a statistically significant difference only for the mean and absolute errors. On average, the variability was comparable to the absolute error, and the error gain approximated unity indicating a slight contraction of the perceived position, although individual cases exhibited more marked contraction or expansion phenomena. In other words, some individuals exhibited an over/underestimation of the amplitude of the perceived flexion/extension administered during the test.

Table 3 reports the wrist sense of position parameters measured in 6 patients after stroke. In accordance with the Fugl-Meyer Sensory subsection, three patients had proprioceptive deficits. Only two of them exhibited increased AE, VE, and reduced EG. The values measured in the third patient were slightly higher than those obtained in healthy individuals. Single subject analysis showed that EG was significantly different (*p* < 0.05) from that measured for the same arm in healthy subjects. Conversely, patients without proprioceptive deficits scored values quite similar to those of the healthy group.

**Figure 2:**
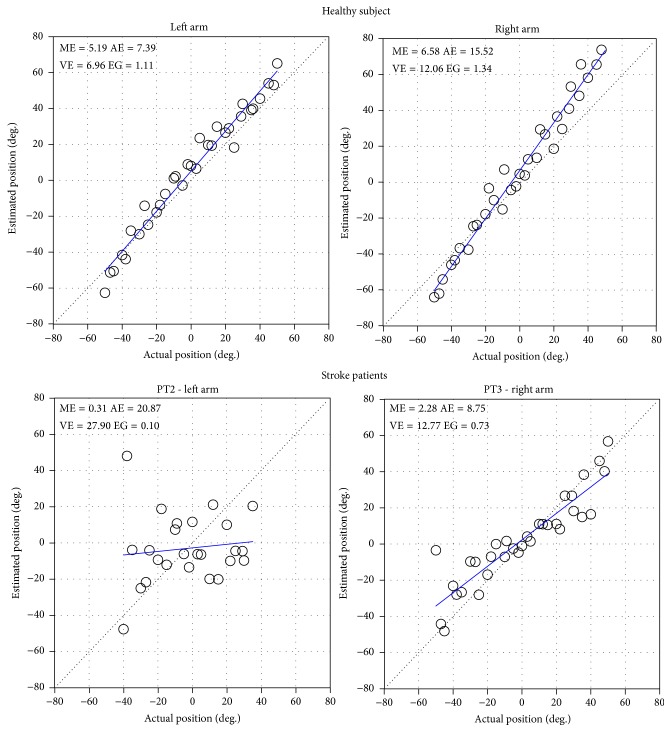
Plot (circle markers) and regression line (solid line) of the actual versus estimated positions in a healthy subject and in a left-impaired patient after stroke with proprioceptive deficits (PT2), and a right-impaired patient after stroke without proprioceptive deficits (PT3). The dotted line represents the identity line.

Figure 2 reports the plot of actual versus estimated positions and the superimposed regression line, obtained with the device for evaluation of wrist sense of position in a 69-year-old healthy subject, a 48-year-old left-impaired patient after stroke with proprioceptive deficits (PT2), and a 51-year-old right-impaired patient after stroke without proprioceptive deficits (PT3). Only PT2 has a pattern that is clearly different from the others.

**Figure 3:**
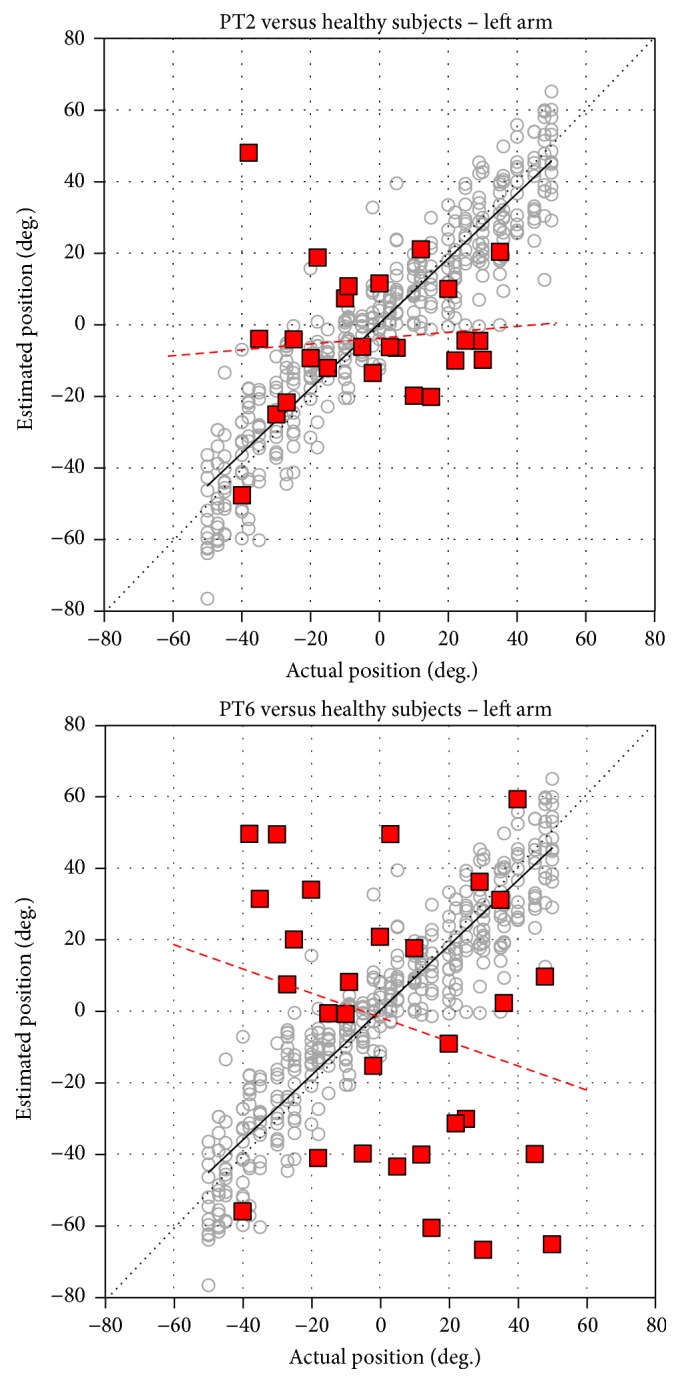
Plot and regression lines of the actual versus estimated positions in the group of healthy subjects (grey circle markers, black solid line) and in two left-impaired patients after stroke with proprioceptive deficits (PT2, PT6; red square markers, red dashed line). The dotted line represents the identity line.

Figure 3 reports the pattern obtained in two of 3 patients with proprioceptive deficits and that for the whole group of healthy subjects. The comparison of their respective regression lines clearly shows the different slopes, thus confirming the results of the single subject analysis.

Table 4 presents the average performance parameters and their standard deviations measured before (PRE) and after (POST) training in the 4 stroke patients with proprioceptive deficits who took part in the proprioceptive training protocol. The VA and AMI parameters both increased during training, but the robot assistance parameter (AMI) had higher values, on average, than visual assistance (VA). In other words, our patients in the first instance requested visual assistance because of their erroneous perception of the current position of their hand/arm in the workspace and subsequently requested the robot assistance. The NVA decreased, indicating that at the start of training patients requested for each reaching movement at least one or more activation of visual assistance, whereas at the end of training the assistance was activated only on average once every two reaching tasks.

In two of the four patients, the improvement of the parameters was reflected in an improvement also of the FM-Sensory scale, that is, the training protocol was effective in improving patients' sense of position.

**Figure 4:**
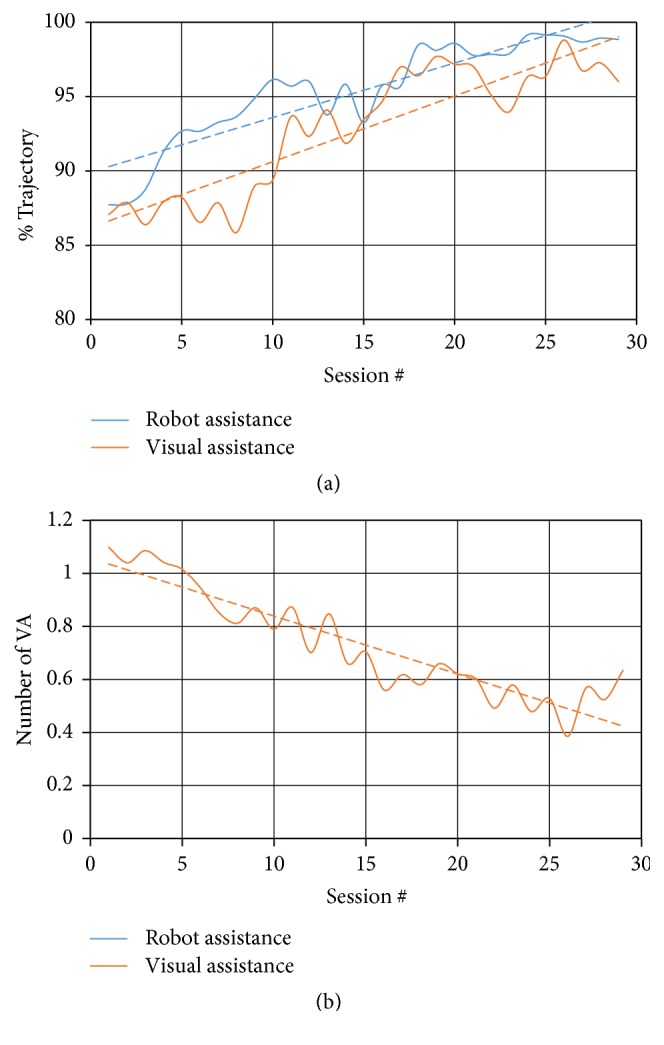
Plot (solid line) and regression line (dashed line) of the three parameters estimating the proprioceptive performance during robot-assisted proprioceptive training of a stroke patient (PTt4). (a) presents the AMI parameter (blue line) and the percentage average visual assistance parameter (VA, orange line). They represent the percentage of trajectory at which, respectively, the robot and visual assistance are activated. (b) presents the normalized number of activations of visual assistance (NVA, orange line).

Figure 4 reports the time course of recovery for the robot assistance (AMI), visual assistance (VA), and NVA parameters during the course of 29 training sessions of a patient (PTt4) admitted to the proprioceptive robot training protocol. Each plotted value represents the mean value scored during a training session. There is a clearly increasing pattern and trend (dashed line obtained by regression analysis) for AMI and VA and a decreasing pattern for NVA.

## 4. Discussion

The main purpose of this study was to validate a system architecture developed for the quantitative assessment and training of the upper limb sense of position in the horizontal plane (proximal and distal evaluation). Our results for healthy and pathological subjects are in line with our previous and other studies in the literature [26, 38, 39, 41, 44].

We showed that the sense of position (mean and absolute error) in healthy subjects varies slightly depending on the side being assessed. This finding, considered with the established importance of the right hemisphere in spatial awareness, points to the possibility of right cerebral dominance in proprioceptive spatial tasks, with a consequent better performance of the left arm [23, 45].

The parameters measured with the device for evaluation of wrist sense of position seem to be able to distinguish healthy from pathologic conditions. The absolute error and variability of the patients with proprioceptive deficits differed consistently from those of both healthy subjects and other patients without proprioceptive deficits. In addition, also the error gain was lower than that of the other subjects. In other words, PT2 and PT6 seemed to exhibit a poor sensitivity in estimating the position of the hand both during extension and flexion movements. Conversely, PT5, on average overestimated the wrist position but his EG parameter was not higher than individual values observed in healthy subjects. In addition, although this patient was classified by the clinician as having an evident sense of position deficit, the proprioception parameters were only slightly different (higher) from those of healthy subjects. This could be ascribed to the fact that his FM-S score accounted for both sense of position and touch deficits. For this reason, a future study with an increased sample size is mandatory to better define normality values and confidence limits for the measured parameters, so as to allow measurement and classification of different levels of impairment. Hence, this study should be considered as a pilot study. In order to fully address the reliability and the normative values of this system, subjects in the future study should also be divided into different classes of age and gender. It has been shown that proprioceptive performance differences in position and motion sense clearly exist between the young and elderly [22], proprioceptive deficits in the elderly being related to their general age-related decline which may impact several sensorimotor tasks. In our study, the mean age of healthy subjects and patients was quite similar but people exhibiting proprioceptive deficits were slightly younger than controls, so single subject comparison should ideally have been carried out on age-matched groups. The patients involved in the training protocol with the shoulder-elbow manipulator exhibited an improved performance after training both in sensory (VA and NVA) and in motor (AMI) parameters. Also in this case, further studies with larger sample size are needed to confirm this encouraging result. In a preliminary study, we administered our training protocol to a small group of healthy subjects (data not reported), but after only a few reaching trials they demonstrated they were able to complete the task without visual and motor assistance.

Finally, no study so far has verified if training of proximal joints can elicit changes in distal proprioception. We believe that the developed architecture is suitable for the assessment of the relationship of changes of proximal and distal parameters, allowing the implementation of specific studies for this purpose.

## 5. Conclusions

The system and the new training protocol we have developed seems to be able to quantitatively evaluate upper limb sense of position at the wrist joint and the proximal changes occurring during training. In addition, it can distinguish healthy from pathological conditions thanks to a set of quantitative parameters. This system could be employed to detect changes in sense of position of patients with sensory deficits after stroke and could enable the implementation of novel training approaches and upper limb rehabilitation protocols specifically devoted to the recovery of normal proprioception.

## Figures and Tables

**Table 1:** Characteristics of healthy individuals and patients after stroke. Continuous variables are reported as mean ± standard deviation, the Ashworth scale as median (interquartile range).

Patient characteristics	Healthy subjects (*n* = 12)	Stroke patients (*n* = 10)
Age (years)	61.5 ± 2.2	60.8 ± 13.8
Sex	3F/9M	3F/7M
Handedness (right/left)	12/0	9/1
Time since acute event (months)		2.5 ± 3.7
Fugl-Meyer motor (0–66 range)		31.8 ± 18.0
Fugl-Meyer sensory (0–12 range)		9.5 ± 2.2
Impaired arm (left/right)		4/6
Type of stroke (hemorrhagic/ischemic)		1/9
Ashworth-shoulder		0 (0)
Ashworth-elbow		0.5 (0-1)
Ashworth-wrist		0 (0)

**Table 2:** Results of wrist proprioception assessment parameters in healthy subjects.

ID	Left arm				Right arm			
	ME (deg)	AE (deg)	VE (deg)	EG (a.u.)	ME (deg)	AE (deg)	VE (deg)	EG (a.u.)
HS1	5.19	7.39	6.96	1.11	6.58	10.52	12.06	1.34
HS2	−6.32	8.14	8.00	1.18	14.96	15.56	8.38	1
HS3	−0.11	6.35	7.93	1.04	5.71	8.64	10.94	0.93
HS4	−1.44	6.64	8.53	0.91	0.16	4.15	5.23	0.94
HS5	4.46	9.66	10.55	0.70	5.26	9.97	11.4	0.67
HS6	−6.33	11.15	13.81	0.69	15.04	15.95	10.73	1.18
HS7	0.04	10.64	13.20	0.64	7.34	8.93	8.6	0.74
HS8	2.93	6.75	7.87	1.02	15.45	17.41	16.45	1.39
HS9	2.62	8.52	9.46	1.21	6.57	9.01	8.78	1.03
HS10	−0.04	8.95	11.01	0.74	6.64	10.38	11.51	0.71
HS11	−4.98	8.04	7.82	0.91	12.96	15.45	14.27	0.62
HS12	8.42	12.24	13.78	0.74	6.32	10.65	11.44	0.94
*Mean*	*0.37*	*8.71*	*9.91*	*0.91*	*8.58* ^*∗∗*^	*11.39* ^*∗*^	*10.82*	*0.96*
*sd*	*4.66*	*1.89*	*2.51*	*0.20*	*4.84*	*3.90*	*2.90*	*0.25*

a.u.: arbitrary units; left versus right comparison: ^*∗*^*p* < 0.05, ^*∗∗*^*p* < 0.001.

**Table 3:** Results of wrist proprioception assessment parameters and Fugl-Meyer score in 6 stroke patients who underwent only the distal evaluation protocol.

ID	Arm	ME (deg)	AE (deg)	VE (deg)	EG (a.u.)	FM-S (0–12)	FM-M (0–66)
PT1	Right	−0.40	6.72	8.67	1.02	12	31
PT2	Left	−0.31	20.87	27.90	0.10	10	34
PT3	Right	2.28	8.75	12.77	0.73	12	14
PT4	Right	3.77	9.97	12.63	0.73	12	24
PT5	Right	−4.51	13.98	16.18	1.22	8	24
PT6	Left	−8.76	42.77	51.87	−0.33	6	65

a.u.: arbitrary units; FM-S/M: Fugl-Meyer sensory/motor sections.

**Table 4:** Proximal proprioceptive performance parameters and Fugl-Meyer score evaluated before and after training in 4 stroke patients who underwent the robot-assisted proprioceptive training protocol.

ID	Before training					After training				
	AMI (%)	VA (%)	NVA (a.u.)	FM-S	FM-M	AMI (%)	VA (%)	NVA (a.u.)	FM-S	FM-M
PTt1	99.22	91.08	0.78	7	58	99.87	95.19	0.37	7	58
PTt2	81.97	55.10	2.67	9	37	100.00	97.50	0.60	9	49
PTt3	77.20	67.56	1.26	7	8	97.75	104.59	1.36	8	9
PTt4	87.78	87.89	1.04	11	23	98.93	97.28	0.52	12	28
*Mean*	*86.54*	*75.41*	*1.44*			*99.14*	*98.64*	*0.71*		
*Sd*	*9.50*	*17.08*	*0.84*			*1.04*	*4.10*	*0.44*		

a.u.: arbitrary units; FM-S/M: Fugl-Meyer sensory/motor sections.
